# Integrated Laparoscopic Management of Parastomal and Midline Incisional Double Hernias: A Staged Approach Including Hartmann's Reversal and Hernia Repair

**DOI:** 10.7759/cureus.60470

**Published:** 2024-05-16

**Authors:** Aina Kunitomo, Shunichiro Komatsu, Tatsuki Matsumura, Yasuyuki Fukami, Tsuyoshi Sano

**Affiliations:** 1 Department of Gastroenterology, Aichi Medical University Hospital, Nagakute, JPN

**Keywords:** double hernias, staged surgery, parastomal hernias, incisional ventral hernia, laparoscopic reversal hartmann's procedure

## Abstract

Parastomal hernia (PH) following Hartmann’s procedure is a common late-term complication and is often combined with an incisional hernia (IH). The surgical treatment for double hernias with an end colostomy is complex and challenging. We present a 54-year-old woman with an end colostomy and combined hernias (PH and midline IH) after an emergency Hartmann’s procedure for diverticular perforation of the sigmoid colon underwent staged surgery. First, laparoscopic Hartmann’s reversal (LHR) and PH repair with primary suture were performed. Ten months later, "intraperitoneal onlay mesh repair (IPOM) plus" methods were implemented for IH repair. Both surgeries were successfully conducted using a laparoscopic approach, and no evidence of hernia recurrence has been observed in the 12 months after the second surgery. This case report provides valuable insights into the surgical strategy for double hernias with an end colostomy.

## Introduction

Parastomal hernia (PH) is a common late-term complication of Hartmann’s procedure [1], with a reported incidence of up to 48% [2]. Although the ideal treatment for PH is to restore bowel continuity with stoma closure (Hartmann's reversal (HR)), this requires major surgery and is associated with high morbidity and mortality rates [3]. Furthermore, a PH and an incisional hernia (IH) are often present together [4], forming a complex configuration of the hernia orifices. Thus, the surgical procedure for double hernias with an end colostomy is complex and challenging.

Herein, we present a case involving simultaneous parastomal and midline IHs following an emergency Hartmann's procedure. HR with primary closure of the PH orifice, followed by second-stage repair of the midline IH using the “intraperitoneal onlay mesh repair (IPOM) plus” method [5], was planned. Both surgeries were successfully performed using a laparoscopic approach.

## Case presentation

**Figure 1:**
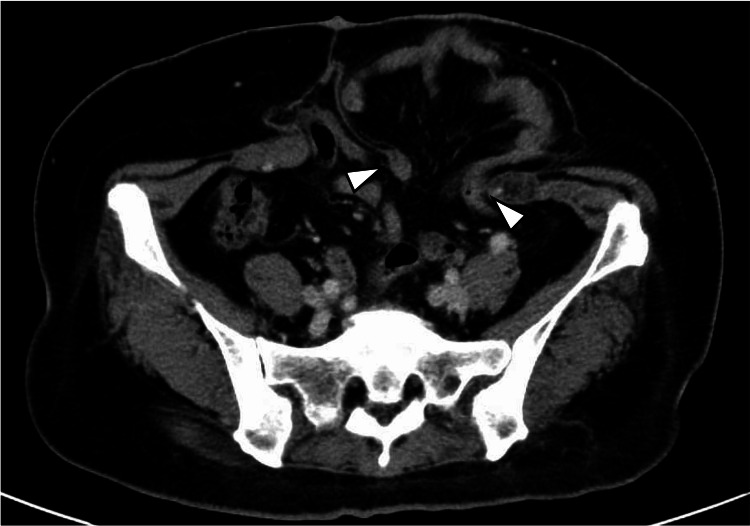
Parastomal hernia Contrast-enhanced computed tomography revealed a parastomal hernia with an orifice of 8.0x4.5 cm. Small intestinal prolapses were evident in the hernia (arrowheads).

**Figure 2:**
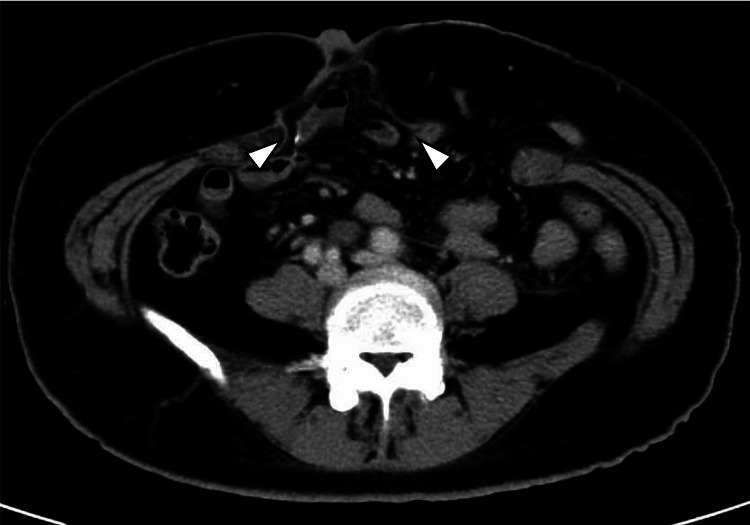
Abdominal midline incisional hernia Contrast-enhanced computed tomography revealed an abdominal midline incisional hernia with an orifice of 15x8.0 cm. Small intestinal prolapses were evident in the hernia (arrowheads).

A 54-year-old woman underwent an emergency Hartmann's procedure due to diverticular perforation of the sigmoid colon at a tertiary hospital nearby. She has been obese since that time, double hernias at the ostomy and midline incisional sites had developed gradually during the eight months after the surgery, and could not be treated at the other hospital. She was referred to our hospital to undergo surgical interventions to remove the stoma and repair the hernias. At the initial visit, the patient was diagnosed as obese with a body mass index of 28.5 kg/m^2^ (weight: 73.0 kg; height: 160.0 cm). Contrast-enhanced computed tomography (CT) revealed the presence of a PH and a midline IH, with prolapse of the small intestine into each hernia (Figures 1, 2, respectively).

The sizes of the hernia orifices for the PH and midline IH were 8.0x4.5 cm and 15x8.0 cm, respectively. We planned staged laparoscopic surgery to treat these hernias as follows: HR to restore intestinal continuity with closure of the PH orifice without using prostheses, and later, repair of the IH using the “IPOM plus” method.

**Figure 3:**
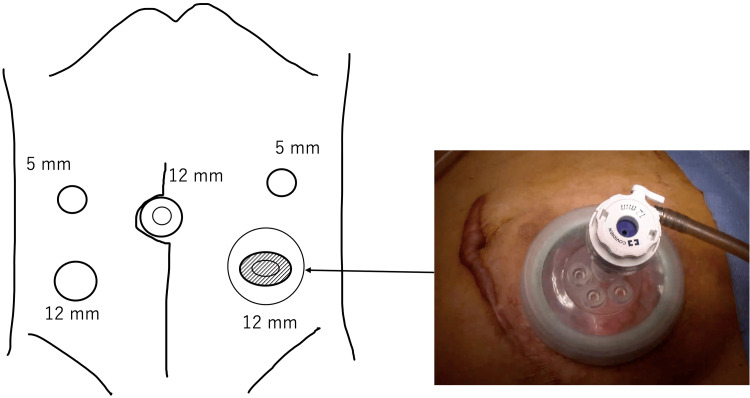
The first laparoscopic access port In the first surgery of a laparoscopic Hartmann’s reversal procedure, a wound retractor (Smart Retractor®, TOP Corporation, Japan) was attached at the site of the removed colostomy and covered by the supplied cap with a trocar; this provided the first laparoscopic access to the abdominal cavity.

For the first surgery, the laparoscopic HR (LHR) procedure was modified from that described previously [6]. The colostomy was temporarily closed and completely detached from the abdominal wall. The colon was then returned to the peritoneal cavity. A wound retractor (Smart Retractor®, TOP Corporation, Japan) was attached at the site of the removed colostomy and covered by the supplied cap with a trocar, providing the first laparoscopic access to the abdominal cavity. Under the pneumoperitoneum, residual ports were added using the typical method of rectal resection (Figure 3).

There were moderate adhesions involving the abdominal wall, the greater omentum, and the small intestine. After meticulous adhesiolysis, the left colon and remaining rectum were mobilized to ensure end-to-end anastomosis. The rectal stump was identified and resected using a laparoscopic linear stapler (Powered ECHELON FLEX®7, Ethicon, Tokyo, Japan). The specimen was retrieved through the stoma incision. The colonic stump was reintroduced into the abdominal cavity after the insertion of a circular anvil (ECHELON CIRCULAR® stapler, Ethicon, Tokyo, Japan). The double-stapling technique anastomosis was performed laparoscopically. The fascia of the stoma site was then closed using absorbable sutures (0-PDS, Ethicon, Tokyo, Japan), whose tension was released by the presence of the midline IH. This was followed by negative pressure wound therapy. The operation time was 413 minutes, and the blood loss was 59 g. The postoperative course was uneventful except for seroma formation at the site of stoma closure. The patient was discharged on postoperative day (POD) 13.

**Figure 4:**
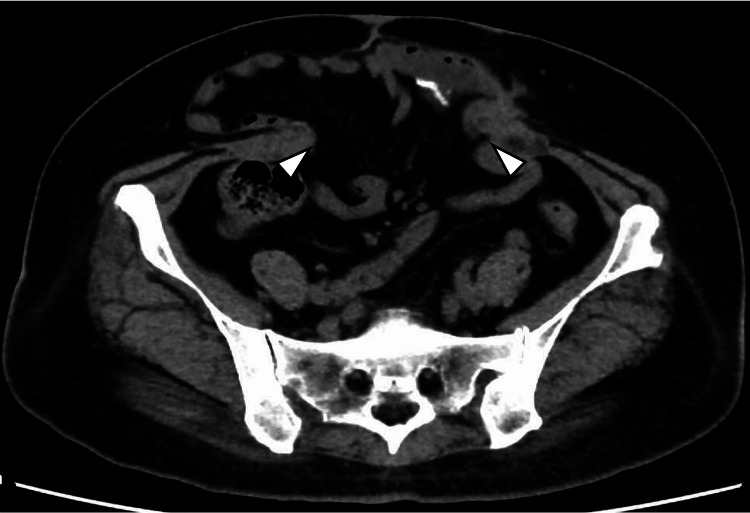
Abdominal midline incision hernia after the first surgery Contrast-enhanced computed tomography scans before the second surgery showed the existing abdominal midline incision hernia with an orifice of 18x11 cm, without recurrence of the parastomal hernia (arrowheads).

The patient had been instructed to lose approximately 5% of her body weight before the second surgery and the goal of weight loss to 69.5 kg was achieved by 10 months after the first intervention. At this time, enhanced CT showed that the size of the hernia orifice in the residual midline IH was 18x11 cm and recurrence of the PH was not observed (Figure 4).

For the second surgery, the laparoscopic “IPOM plus” method was performed after an interval of 10 months, as described previously [5]. The first port was placed at Palmer’s point using the optical method because major adhesions were suspected in the abdominal cavity. There were in fact severe adhesions involving the abdominal wall and small intestine. Ports were added in order from the upper to lower abdomen, as needed, during the adhesiolysis procedure. After the abdominal wall was free from adhesions, the fascia of the hernia orifice was sutured and closed with eight stitches. Then, a mesh was inserted in the abdominal cavity and secured by the double crown technique, using a Capsure® (Bard, Tokyo, Japan). The mesh used was a 25x20 cm Symbotex™ round mesh (Medtronic Japan, Tokyo, Japan) coated with collagen to prevent adhesions and was sufficiently large to overlap the hernia orifice. The operation time was 154 minutes, and the blood loss was 25 g. The postoperative course was uneventful, and the patient was discharged on POD 6. No evidence of hernia recurrence and no complaints of symptoms have been found in the 12 months after the second surgery.

## Discussion

The laparoscopic approach for HR has been increasingly used, with reportedly reduced morbidity and mortality rates compared to open surgery [7]. However, there is a notable absence of reports on LHR surgeries specifically performed for the treatment of PH. Additionally, there is a lack of established guidelines or evidence-based literature for the treatment of double hernias involving both PH and midline IH. Our report describes the novel attempt of combining LHR with hernia repair in a patient who underwent emergency surgery for perforated sigmoid colon diverticulosis. The additional IH was subsequently repaired in a staged manner, and the sequential series were completed using a minimally invasive approach.

Synchronous closure of colostomy and ventral hernia repair following Hartmann’s procedure is associated with an increased risk of postoperative complications [8,9]. Concerns about mesh infection suggest that prosthetic repair of hernias with concomitant stoma closure should be avoided [8]. Thus, we opted for staged surgery in this case of ventral hernia with an end colostomy. A standard procedure was employed for the midline IH, with a synthetic mesh to reduce the recurrence rate.

Another critical aspect of this case was the management of large and complex defects of the fascia. Typically, two hernias are repaired separately using appropriately sized meshes for each defect. However, preventing hernia recurrence requires a large prosthesis to overlap at least 5 cm from the hernia orifice in all directions [10]. If two large meshes were used for double hernias, they would pile up, making it technically difficult to spread and fix the sheets on the abdominal wall. Our approach, involving primary suture repair for the PH in the first surgery and subsequent IH treatment with a single mesh, simplifies prosthetic repair and may be regarded as a reasonable treatment strategy.

## Conclusions

The surgical treatment for double hernias with an end colostomy and IH is complex and challenging. Staged surgery reduces the risk of infection and simplifies the hernia repair procedure. Furthermore, the repeated laparoscopic approaches, avoiding a large abdominal incision, may contribute to reducing the risk of further hernia development and recurrence. This case report provides valuable insights into the surgical strategy for double hernias with an end colostomy.
